# Synaptic Dysfunction in Prion Diseases: A Trafficking Problem?

**DOI:** 10.1155/2013/543803

**Published:** 2013-11-28

**Authors:** Assunta Senatore, Elena Restelli, Roberto Chiesa

**Affiliations:** Dulbecco Telethon Institute and Department of Neuroscience, IRCCS-Istituto di Ricerche Farmacologiche “Mario Negri,” Via G. La Masa 19, 20156 Milano, Italy

## Abstract

Synaptic dysfunction is an important cause of neurological symptoms in prion diseases, a class of clinically heterogeneous neurodegenerative disorders caused by misfolding of the cellular prion protein (PrP^C^). Experimental data suggest that accumulation of misfolded PrP^C^ in the endoplasmic reticulum (ER) may be crucial in synaptic failure, possibly because of the activation of the translational repression pathway of the unfolded protein response. Here, we report that this pathway is not operative in mouse models of genetic prion disease, consistent with our previous observation that ER stress is not involved. Building on our recent finding that ER retention of mutant PrP^C^ impairs the secretory trafficking of calcium channels essential for synaptic function, we propose a model of pathogenicity in which intracellular retention of misfolded PrP^C^ results in loss of function or gain of toxicity of PrP^C^-interacting proteins. This neurotoxic modality may also explain the phenotypic heterogeneity of prion diseases.

## 1. Introduction

Prion diseases, also known as transmissible spongiform encephalopathies, are progressive and invariably fatal degenerative disorders of the central nervous system (CNS) that affect humans and other animals [1]. Creutzfeldt-Jakob disease (CJD), Gerstmann-Sträussler-Scheinker (GSS) syndrome, and fatal familial insomnia (FFI) are the most common forms in humans; scrapie of the goat and sheep, bovine spongiform encephalopathy (BSE), and chronic wasting disease of deer and elk are the best-known examples of prion zoonoses [2]. Widespread neuronal loss, astrocytosis, spongiform change (vacuolation of the neuropil in the gray matter), and in some cases amyloid plaques are key neuropathological findings in prion diseases, which in humans usually present with loss of motor coordination and other motor abnormalities, dementia, and neurophysiological deficits [3].

Similarly to other progressive neurodegenerative disorders, such as Alzheimer's and Parkinson's disease, frontotemporal dementia, and the tauopathies, prion diseases can arise sporadically or be genetically inherited; however, they can also be acquired by infection [4]. This is dramatically illustrated by kuru, a prion disease of the Foré-speaking people of Papua New Guinea, which used to be transmitted among women and children by ritual cannibalism [5]. Other forms transmitted by infection are variant CJD (vCJD) due to consumption of BSE-infected meat products and iatrogenic CJD in recipients of cadaveric sources of human growth hormone or dura mater grafts or blood transfusions from asymptomatic donors who subsequently died from vCJD [6, 7].

The infectious agent (prion) is scrapie prion protein (PrP^Sc^) [8]. This is a conformationally altered isoform of the cellular prion protein (PrP^C^), a glycosylphosphatidylinositol (GPI)-anchored cell surface glycoprotein of uncertain function expressed at the highest level by neurons in the CNS [9–11]. Like most membrane-associated proteins, PrP^C^ is cotranslationally translocated into the endoplasmic reticulum (ER), where it undergoes oxidative folding and facultative N-linked glycosylation. After transit in the Golgi, PrP^C^ is delivered to the cell surface, where it resides in lipid rafts. Cell surface PrP^C^ can be released into the extracellular space or internalized to an endosomal compartment, from which it is either recycled to the plasma membrane or diverted to lysosomes for degradation [12].

PrP^C^ and PrP^Sc^ have identical amino acid sequences but distinct conformations and biochemical properties. PrP^C^ has a predominant *α*-helix content and is soluble in detergents and protease-sensitive. In contrast, PrP^Sc^ is rich in *β*-sheets, tends to form detergent-insoluble aggregates, and shows variable degrees of resistance to proteinase-K (PK) digestion [13, 14].

PrP^Sc^ propagates by imprinting its aberrant conformation onto endogenous PrP^C^ molecules [8]. This conversion starts on the cell surface [15] and proceeds within the endocytic compartment [16, 17]. It probably involves a process of nucleated polymerization in which oligomers of PrP^Sc^ serve as seeds that recruit and stabilize abnormal conformations of PrP^C^, followed by fragmentation of the PrP^Sc^ polymers into new propagation-competent oligomers [18, 19].

Genetic prion diseases, including familial CJD, GSS, FFI, and PrP-cerebral amyloid angiopathy (PrP-CAA) are linked to point mutations or insertions in the *PRNP* gene encoding PrP^C^ [20]. These diseases are thought to arise because of an intrinsic tendency of the mutant PrP^C^ molecules to misfold and aggregate, eventually acquiring the PrP^Sc^ structure. Sporadic prion diseases, including the majority of CJD cases, sporadic fatal insomnia, and the recently described variably protease-sensitive prionopathies [21], are believed to be due to spontaneous misfolding of wild-type PrP^C^, at a low frequency or to rare somatic *PRNP* mutations.

Prion diseases vary widely in their clinical presentation. CJD is a subacute spongiform encephalopathy mostly involving the cerebral cortex, striatum, and cerebellum and recognized clinically by dementia and motor abnormalities. FFI is characterized clinically by sleep alterations and autonomic dysfunction and neuropathologically by severe degeneration of the anterior ventral and mediodorsal nuclei of the thalamus [22]. GSS is a slowly progressive ataxia with PrP amyloidosis mainly in the cerebellum and basal ganglia. PrP-CAA is a slowly progressive dementia with PrP-amyloid deposits in blood vessels of the CNS [23, 24].

The reason for this variability is not known. Brain tissues from patients with different prion diseases contain pathological forms of PrP with variable degrees of protease resistance and/or distinct PK cleavage sites, suggesting that different conformational isoforms of PrP may have specific neurotoxic properties.

Only recently have we begun to understand how abnormally folded PrP causes neuronal dysfunction and degeneration. Experimental evidence indicates a dissociation between prion infectivity and pathogenicity and suggests that abnormal forms of PrP, structurally different from PrP^Sc^, are the actual trigger of the neurodegenerative process [25]. Nerve endings are the initial targets of the toxic PrP species, which perturbs normal synaptic function and morphology. Beyond this step, when functional recovery is still possible [26], synaptic loss and neuronal death are irreversible stages of the pathogenic process.

## 2. Starting from the End: Neuronal Death in Prion Diseases

The observation that neurodegeneration in prion diseases occurred in the absence of a typical tissue inflammatory response [27, 28] suggested the involvement of programmed cell death (PCD), rather than necrosis. PCD is an active process requiring activation of gene expression and protein synthesis and is morphologically and biochemically distinguishable from necrosis. There are many types of PCD, but only apoptosis and autophagy have been consistently reported in natural and experimental prion diseases.

### 2.1. Apoptosis

Apoptosis is morphologically characterized by shrinkage of the cell, condensation of the chromatin, blebbing of the plasma membrane, and fragmentation of the nucleus, without significant morphological alterations of other subcellular organelles. In the endstage, small membrane-bound cell fragments (apoptotic bodies) are formed, that are rapidly ingested by phagocytic cells without inducing an inflammatory reaction. Biochemically, apoptosis involves internucleosomal cleavage of genomic DNA and in mammals is regulated by the Bcl-2 (B-cell lymphoma protein 2) family of proteins, Apaf-1 (apoptotic protease-activating factor 1), and the cysteine protease caspase family [29].

The first clue to apoptosis in prion disease was nuclear fragmentation and internucleosomal DNA cleavage in primary neurons exposed to PrP106-126, a synthetic peptide used to model prion-induced neuropathology [30]. Analysis of brains from scrapie-affected sheep, CJD and FFI patients, and experimentally prion-infected rodents identified cells with fragmented nuclei, DNA cleavage, and caspase activation, confirming the involvement of PCD [31–45]. In addition, transgenic Tg(PG14) mice expressing a mutant PrP carrying a nine-octapeptide repeat insertion associated with a genetic prion disease showed massive apoptosis of cerebellar granule neurons (CGNs) [46]. Finally, morphological and biochemical features of apoptosis were seen in hypothalamic GT1 cells, primary CGNs, and cerebellar organotypic cultures infected with scrapie [47–49].

Several studies investigated whether blocking the apoptotic program could prevent or ameliorate prion pathology. Transgenic overexpression of the antiapoptotic Bcl-2 protein or targeted deletion of the proapoptotic gene Bax (Bcl-2-associated X protein) did not prevent neuronal loss and neurological disease in prion-infected mice [50, 51], neither did genetic ablation of caspase-12, a proposed mediator of ER stress-induced cell death [52]. Bax deletion rescued CGNs in Tg(PG14) mice but did not prevent the synaptic degeneration and the progressive neurological disease that develop in this model [53]. These results indicated that targeting Bcl-2 family-dependent or ER stress-related apoptotic pathways was not enough to prevent neurodegeneration and suggested that additional (or alternative) mechanisms could be operative in prion diseases leading to synaptic loss and neuron demise.

### 2.2. Autophagy

Macroautophagy (hereafter referred to as autophagy) is a physiologically regulated catabolic pathway that despatches cytoplasmic material, like long-lived proteins and organelles, to the lysosomes for degradation. It is a multistep process in which part of the cytoplasm is initially enclosed in a double-membraned structure to form the autophagosome, also called autophagic vacuole. The autophagosome then fuses with lysosome to form an autolysosome, where the captured material is degraded by lysosomal hydrolases. Autophagosomes can also fuse with early endosomes or multivesicular bodies (late endosomes) to form amphisomes, which then fuse with lysosomes for degradation [54].

Autophagic cell death is presumed to result from excessive levels of cellular autophagy. Morphologically there is degradation of organelles with preservation of cytoskeletal elements until late stages and, like apoptosis, it does not instigate a tissue inflammatory response. Recent data point to a close interplay between autophagy and apoptosis, with the former acting as an inhibitor of the apoptotic program or occurring upstream of apoptosis [55, 56].

A number of studies have brought to light a possible role of autophagy in prion diseases. Abundant autophagic vacuoles and multivesicular bodies were seen in synaptic terminals, neuritis, and neuronal cell bodies in the CNS of prion-infected rodents, CJD, GSS, and FFI patients [57–64]. However, in contrast to a putative disease-promoting activity of autophagy, its pharmacological induction slightly prolonged survival of prion-infected mice [65–67] and significantly delayed the onset and progression of neurological illness in a Tg mouse model of GSS [68]. This beneficial effect was attributed to enhanced clearance of the pathological PrP isoform [65, 68, 69]. A recent report confirmed an increase in autophagic flux in prion-infected cells but found that PrP^Sc^ undergoes lysosomal degradation independently of the autophagic route [70].

Additional studies are necessary to clarify whether autophagy serves a protein quality-control function against misfolded PrP and if its failure or overactivation contributes to neurodegeneration [71]. It will be important to identify the signaling pathways that activate autophagy in prion disease and test the effect of genetic interference in animal models. It will also be essential to investigate the interplay between autophagy and apoptosis, as therapeutic inhibition of PCD depends on understanding how one process controls the other. However, blocking neuronal death might not be sufficient to halt prion disease. Growing evidence, in fact, suggests that synaptic failure, rather than the actual death of neurons, is the primary cause of neurological dysfunction in prion disorders.

## 3. Synaptopathy in Prion Diseases: Correlation between Symptoms and Synaptic Failure

The relation between synaptic pathology and neurological deficits has been extensively studied in mice intrahippocampally injected with Me7 or 87V prions. In these models, there is a progressive decrease in the number of synapses in the *stratum radiatum* with degeneration of the presynaptic compartment and loss of dendritic spines, well before death of CA1 pyramidal neurons [72–76]. Concomitant with this initial synaptic pathology, there are abnormalities in hippocampal synaptic plasticity, which parallel alterations in spontaneous ethological behaviors such as open field activity, burrowing, and nesting [77, 78]. A similar pathological sequence is seen in mice intrahippocampally injected with RML prions, in which defects in presynaptic hippocampal function and degeneration of synapses parallel deficits in recognition memory, burrowing, and nesting and precede loss of pyramidal cells [79, 80]. Thus synaptic dysfunction and degeneration are important determinants of the early behavioral abnormalities in prion-infected mice.

Disruption of synaptic connectivity is an important correlate of symptomatology also in human prion diseases. Neuropathological analyses in humans are of necessity restricted to the terminal phase of the illness when there is often extensive loss of neurons in addition to synaptic degeneration. However, cases of genetic prion disease linked to octapeptide repeat insertions have been described that show widespread synaptic loss but preservation of nerve cells, supporting the idea that the neurological deficits correlate with loss of neuronal processes rather than cell bodies [81]. This is corroborated by experiments in Tg(PG14) mice, indicating that synaptic disruption is the major determinant of neurological illness (see Section 5) [53, 82].

What causes synaptic failure in prion disease? Abnormal PrP deposition is extracellular in most forms of prion disease, often occurring as diffuse protease-resistant “synaptic-like” deposits in perineuronal structures throughout the neuropil [83]. Therefore, a common assumption is that synaptic loss is due to a direct toxic effect of accumulated PrP. In the Me7 model, neither the magnitude nor the spatial pattern of PrP^Sc^ deposition correlates with the number of synapses lost [73, 75]. Moreover, in 87V-infected mice, alterations in synaptic morphology in the hippocampus occur before PrP^Sc^ deposition [39]. In Tg(PG14) mice, protease-resistant PrP, as detected by immunocytochemistry, accumulates in the molecular layer of the cerebellum in a synaptic-like pattern [46]; however, immunoelectron microscopy demonstrates that PG14 PrP accumulations are not truly synaptic in their localization [84]. Thus, in both infectious and genetic models, synaptic degeneration cannot be readily explained by a toxic effect of deposited PrP.

It may be argued that soluble rather than deposited forms of PrP^Sc^ are the actual synaptotoxic species. Monomers and soluble oligomers of recombinant PrP have been generated *in vitro* that are toxic to neurons in culture and after intracerebral injection in mice [85–87]. However, it remains to be seen whether similar forms of soluble PrP are generated in prion disease and play any role in synaptic dysfunction.

An alternative explanation is that synaptic failure is the consequence of PrP^C^ misfolding within neurons. Recent data point to a crucial role of PrP accumulation in the ER.

## 4. An ER Stress-Mediated Mechanism of Synaptic Dysfunction in Prion-Infected Mice

**Figure 1:**
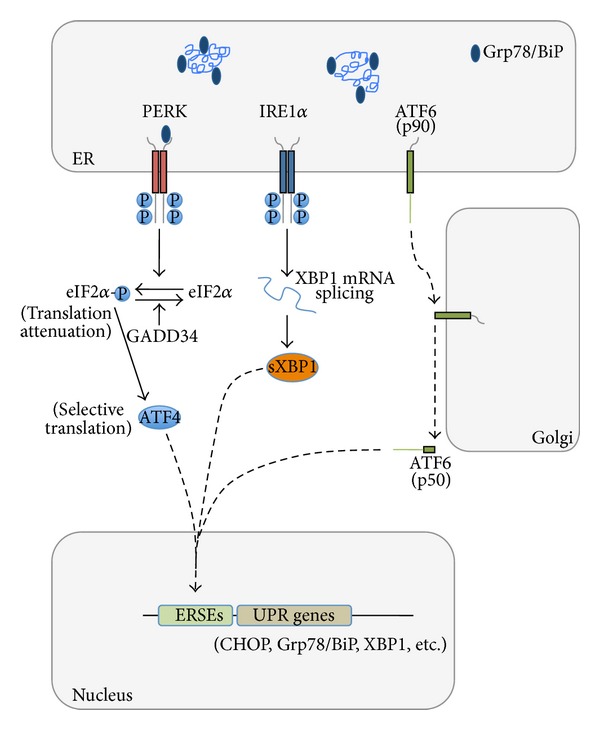
UPR signaling pathways in mammalian cells. The UPR is mediated by three ER-resident transmembrane proteins that sense ER stress through Grp78/BiP binding/release to their luminal domains and/or through direct interaction with unfolded proteins. The kinase PERK (double-stranded RNA-activated protein kinase-like ER kinase) is activated by dimerization and phosphorylation. Once activated, it phosphorylates eIF2*α* (eukaryotic translation initiation factor 2). This inhibits protein translation, reducing the overload of misfolded proteins. This pathway also selectively enhances translation of ATF4 (activating transcription factor 4) that induces the expression of CHOP. In ER-stressed cells, IRE1*α* (inositol-requiring transmembrane kinase and endonuclease) multimerizes and autophosphorylates, setting in motion its RNAse activity. Activated IRE1*α* initiates the unconventional splicing of the mRNA encoding the transcriptional factor XBP1 (X-box-binding protein 1) to produce sXBP1, a more stable form of XBP1 with a potent transactivator domain that enhances transcription of genes involved in protein folding, secretion, and ER-associated degradation. Another ER stress sensor is ATF6 (activating transcription factor 6). This is a type II ER transmembrane protein whose cytosolic domain contains a bZIP transcriptional factor. ATF6 is transported to the Golgi where it is processed within the transmembrane domain to release the cytosolic domain, which translocates to the nucleus and induces expression of the ER chaperone Grp78/BiP and XBP1. GADD34, a protein phosphatase upregulated by the PERK pathway, dephosphorylates eIF2*α* to restore global protein synthesis. ERSEs: ER stress responsive elements.

Moreno et al. discovered a molecular mechanism underlying synaptic failure in RML-infected mice [80]. They found that PrP accumulation in the hippocampus was associated with activation of the translational repression pathway of the unfolded protein response (UPR). The UPR is an adaptive signal transduction cascade that is activated when misfolded proteins accumulate and aggregate in the ER; it involves a tripartite signaling that enhances the folding capacities in the ER, improves misfolded protein disposal through ER-associated degradation, and reduces the rate of protein synthesis and translocation into the ER lumen (Figure 1) [88, 89]. The signal for repression of protein synthesis is triggered by the autophosphorylation of the ER-associated kinase PERK, which phosphorylates the *α* subunit of eukaryotic translation initiation factor 2 (eIF2*α*). This inhibits protein translation, reducing the overload of misfolded proteins. Phosphorylation of eIF2*α* also activates ATF4, a transcription factor that induces expression of CHOP. ATF4 and CHOP cooperate to restore mRNA translation by upregulating target genes encoding functions in protein synthesis [90]. If the adaptive UPR effectively reduces the unfolded protein load, restoration of protein synthesis promotes cell survival. However, if protein synthesis increases before restoration of proteostasis, a signal is activated that promotes apoptotic cell death [90].

Moreno et al. found a progressive increase in PERK and eIF2*α* phosphorylation in the hippocampus of RML-infected mice, in parallel with accumulation of PrP^Sc^ and rising levels of total PrP [80]. They reported a decline in protein translation with a sudden drop in the levels of pre- and postsynaptic proteins, such as the SNARE proteins SNAP-25 and VAMP-2, the NR1 subunit of the N-methyl-D-aspartate receptors (NMDARs), and PSD-95. This was associated with a deficit in hippocampal synaptic transmission and abnormal burrowing behavior. Lentivirally mediated overexpression of GADD34, a specific eIF2*α*-P phosphatase, reduced eIF2*α*-P levels and restored protein synthesis, rescuing the synaptic transmission defect and the behavioral abnormalities. The same effects were seen upon neuron-specific PrP^C^ silencing by RNA interference [80], suggesting that accumulation of misfolded PrP^C^ in the neuronal ER was the proximate cause of UPR and PERK-mediated translational repression. Thus, intraneuronal PrP^C^ misfolding during prion infection would ultimately lead to synaptic failure by reducing the levels of proteins essential for synaptic transmission. Another study suggested that hyperactivation of calcineurin due to calcium release from the stressed ER could also contribute to neuronal dysfunction in prion disease [91].

Our findings in mouse models of genetic prion disease are consistent with the idea that ER retention of misfolded PrP affects synaptic function but that ER stress is not involved.

## 5. Alterations in Voltage-Gated Calcium Channel Activity Underlie the Neurotransmission Deficit Associated with Motor Impairment in Mutant PrP Mice

Tg(PG14) mice develop a progressive neurological illness characterized clinically by ataxia and neuropathologically by cerebellar atrophy due to loss of synaptic endings in the molecular layer and massive apoptosis of CGNs [46, 92]. To test whether blocking the apoptotic program could prevent neurodegeneration and motor dysfunction, we crossed Tg(PG14) with Bax knockout mice. Bax deletion efficiently rescued CGNs but had no effect on the development of ataxia and synaptic loss [53]. This suggested that disruption of synaptic connectivity in the cerebellum was vital in the Tg(PG14) disease and prompted us to test whether abnormalities in neurotransmission could be detected before neurodegeneration, in parallel with the onset of motor dysfunction.

We found that the motor behavioral deficits in Tg(PG14) mice emerged before synaptic loss and were associated with defective glutamatergic neurotransmission in CGNs due to impaired calcium influx through voltage-gated calcium channels (VGCCs) [82]. The same functional changes were seen in CGNs of Tg(CJD) mice that express the mouse PrP homologue of the D178N/V129 mutation linked to genetic CJD and develop motor abnormalities in the absence of granule cell death [82, 93]. Thus, in two different mouse models of genetic prion disease, the onset of motor behavioral abnormalities was dissociated from neuron demise and correlated with defective glutamatergic transmission in CGNs due to alterations in VGCC activity.

## 6. ER Retention of PG14 PrP Is Not Associated with an ER Stress Response

**Figure 2:**
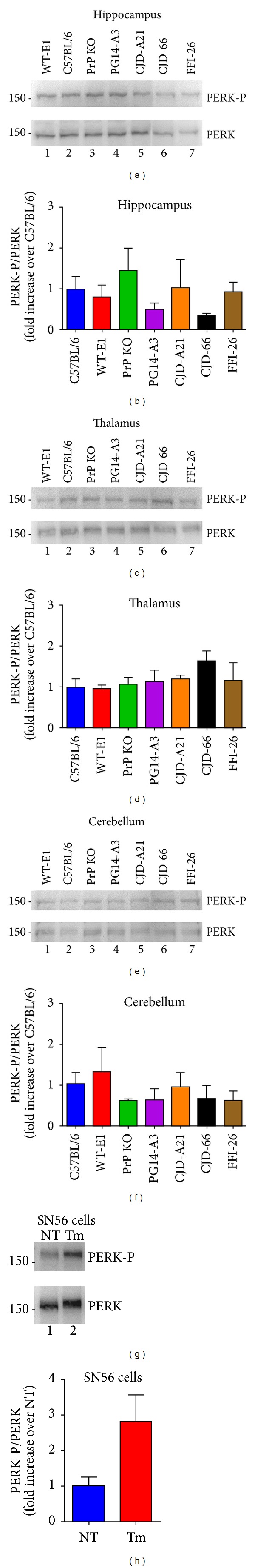
Phosphorylation of PERK is not increased in the brains of mutant PrP mice. Phosphorylation of PERK was evaluated in brain extracts of the following mice: C57/BL6J (PrP level 1X), PrP KO (C57BL/6J/*Prnp* ^0/0^, European Mouse Mutant Archive, Rome, Italy; EM: 01723), Tg(WT-E1^+/+^) overexpressing 3F4-tagged wild-type PrP at ~4X, Tg(PG14-A3^+/−^) expressing 3F4-tagged PG14 PrP at ~1X, Tg(CJD-A21^+/−^) expressing 3F4-tagged D177N/V128 PrP at ~1X, Tg(CJD-66^+/−^) expressing untagged D177N/V128 PrP at ~2.5X, and Tg(FFI-26^+/−^) mice expressing untagged D177N/M128 PrP at ~2.5X. These mice were originally generated on a C57BL/6J X CBA hybrid and then bred with C57BL/6J/*Prnp* ^0/0^ mice ([92, 93] and manuscript in preparation). Proteins were extracted from the hippocampus, thalamus, and cerebellum of mice of the indicated strains/genotype ((a)–(f)) or from SN56 cells ((g) and (h)), using a lysis buffer containing 50 mM Tris, 150 mM NaCl, 2 mM EDTA, 1 mM MgCl_2_, 100 mM NaF, 10% glycerol, 1% Triton X-100, 1% Na deoxycholate, 0.1% SDS, and 125 mM sucrose, supplemented with Phos-STOP and protease inhibitors (Roche) [80]. Protein extracts (50 *μ*g) were analyzed by Western blot with anti-PERK-P and antitotal PERK antibodies (1 : 1000; Cell Signaling) ((a), (c), (e), and (g)). Molecular mass markers are in kilodaltons. Phosphorylation levels were quantified by densitometric analysis of Western blots and expressed as the -fold increase over the level in C57BL/6 mice ((b), (d), (f), and (h)). Tunicamycin (Tm) treated HeLa cells were analyzed at 2 hours as control for UPR activation. Each value is the mean ± SEM of three animals of 300–350 days of age or from three independent cell preparations.

**Figure 3:**
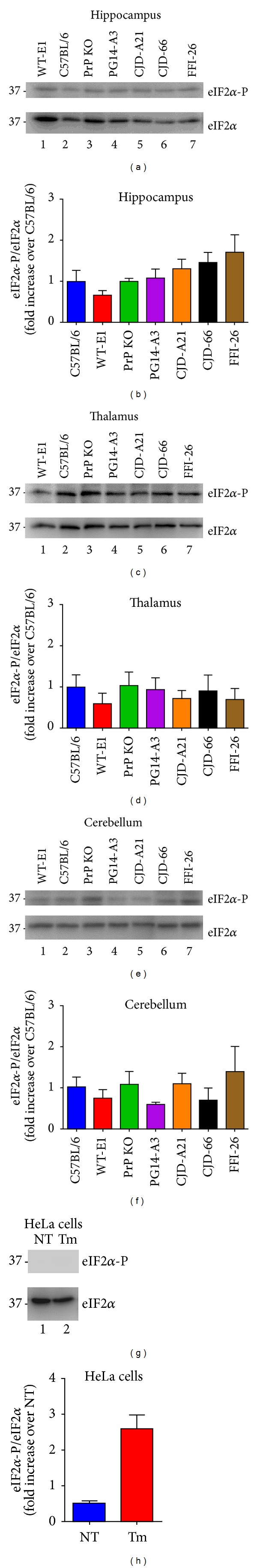
Phosphorylation of eIF2*α* is not increased in brains of mutant PrP mice. The same brain protein extracts (20 *μ*g) as in Figure 2 ((a)–(f)) or lysates of HeLa cells ((g) and (h)) were analyzed by Western blot with anti-eIF2*α*-P and antitotal eIF2*α* antibodies (1 : 1000; Cell Signaling). Molecular mass markers are in kilodaltons. Phosphorylation levels were quantified by densitometric analysis of Western blots and expressed as the -fold increase over the level in C57BL/6 mice ((b), (d), (f), and (h)). Tunicamycin (Tm) treated HeLa cells were analyzed at 2 hours as control for UPR activation. Each value is the mean ± SEM of three animals of 300–350 days of age or from three independent cell preparations.

Analysis of PG14 PrP metabolism and localization in CGNs showed that this mutant misfolds soon after synthesis in the ER, is delayed in its biosynthetic maturation, and accumulates abnormally in this organelle [94–96]. This suggested that intracellular accumulation of mutant PrP might be critical in neuronal dysfunction, possibly due to activation of an ER stress response [25]. However, molecular biology, biochemical and immunohistochemical analyses of brain tissues, and primary CGNs from the mutant mice found no increase in the expression of UPR-regulated genes [97] or activation of the PERK/eIF2*α* translational repression pathway (Figures 2 and 3). There were also no changes in the amounts of synaptic proteins, as the levels of synaptophysin, SNAP-25, the synaptic vesicle fusion protein synaptotagmin I, and the secretory vesicle chaperone CSP*α* were not affected in Tg(PG14) at the onset of the cerebellar deficit [82]. Thus, in contrast to RML-infected mice where alterations in synaptic function correlate with ER stress-induced translational repression [80], the neurotransmission defect in Tg(PG14) mice was not associated with a decrease in protein synthesis as a consequence of ER stress. Moreover, calcineurin activity was decreased rather than induced in the Tg(PG14) cerebellum [98], arguing against an involvement of ER stress-induced calcium release and calcineurin hyperactivation in synaptic dysfunction.

How might the lack of an ER stress response be explained despite demonstrable mutant PrP misfolding and retention in this organelle? A reasonable explanation is that PG14 PrP never accumulates in the ER to a high enough level to trigger the UPR. We did in fact find that although it was delayed in its biosynthetic maturation, PG14 PrP eventually escapes the ER quality control system of the cell and is trafficked to post-ER compartments [94–96].

In the next section, we describe the mechanism by which impaired trafficking of PG14 PrP alters VGCC function. Our studies brought to light an alternative, UPR-independent modality by which intracellular PrP^C^ misfolding affects synaptic proteostasis.

## 7. ER Retention of Mutant PrP Causes Inefficient Synaptic Targeting of VGCCs

How could misfolding of mutant PrP in the ER alter VGCC function? First, we asked whether intracellular PrP retention was responsible for the VGCC defect. We found that PG14 PrP molecules with a deletion in the hydrophobic core (HC) between residues 114 and 121 had less tendency to misfold and accumulate in transport organelles and were more efficiently delivered to the cell surface than their full-length counterparts [99], providing a model for assessing the role of intracellular retention. We compared the effect of HC-deleted and full-length PG14 on neuronal calcium dynamics and found that the calcium response in CGNs expressing HC-deleted PG14 PrP was similar to that of the wild-type controls [82]. This suggested that misfolding and ER retention of mutant PrP were necessary to induce the VGCC defect.

Because our data pointed to a role of intracellular PrP retention, we hypothesized that PG14 PrP interacted with VGCCs in transport organelles, interfering with their trafficking towards the plasma membrane. VGCCs are heteromeric proteins consisting of the pore-forming Ca_V_ *α*_1_ subunit, which governs the biophysical and pharmacological properties of the channel, and the auxiliary *α*_2_ *δ* and Ca_V_ *β* subunits, which regulate the cellular trafficking and activity of Ca_V_ *α*_1_ [100]. Glutamate release from CGNs is mainly governed by P/Q-type channels made of the Ca_V_ *α*_1A_, *α*_2_ *δ*-1, and Ca_V_ *β*_4_ subunit isoforms. The *α*_2_ *δ* subunits play a vital role in intracellular trafficking of the pore-forming Ca_V_ *α*_1_ subunits and boost calcium current amplitude by increasing the number of channels on the cell surface [101, 102]. Thus, retention of *α*_2_ *δ* in secretory organelles due to interaction with mutant PrP could impair VGCC delivery and function at presynaptic sites.

Our studies confirmed this. We found a physical interaction between *α*_2_ *δ*-1 and PrP (both wild-type and mutant) by co-immunoprecipitation, and the two proteins colocalized in transfected cells. We also observed that *α*_2_ *δ*-1 and Ca_V_ *α*_1A_ were weakly expressed on the cell surface and localized intracellularly in mutant PrP-expressing cells, indicating impaired secretory transport. Finally, we found smaller amounts of *α*_2_ *δ*-1 and Ca_V_ *α*_1A_ in cerebellar synaptosomal fractions of Tg(PG14) mice and reduced colocalization with synaptic markers, consistent with inefficient targeting of the channel complex to axonal terminals of granule neurons [82].

Thus, owing to ER retention of mutant PrP, *α*_2_ *δ*-1 accumulates intracellularly, impairing delivery of the VGCC complex to synapses. This negatively affects depolarization-induced calcium influx and glutamate release, leading to alterations of cerebellar synaptic transmission and motor control.

## 8. Other Possible Pathological Consequences of Mutant PrP Interactions in the ER

The observation that the synaptic delivery of VGCCs is impaired in neurons expressing mutant PrP due to interaction with *α*_2_ *δ*-1 suggests that the secretory transport of other PrP-interacting cargoes may also be impaired. Possible candidates are the *α*_2_ *δ*-2 and *α*_2_ *δ*-3 isoforms, which share fairly high sequence identity with *α*_2_ *δ*-1, and have been identified as potential PrP interactors in proteomic screening [103]. Different *α*_2_ *δ* isoforms are expressed in functionally distinct neurons of the brain, so an impairment of their trafficking resulting from sequestration by mutant PrP may affect VGCC function and neurotransmission in different neural circuits, accounting for the complex symptomatology of genetic prion diseases.

Other proteins involved in neurotransmission, whose secretory transport could be altered by mutant PrP, are the NMDARs and *α*-amino-3-hydroxy-5-methyl-4-isoxazolepropionic acid receptors (AMPARs). These ligand-gated ion channels are composed of combinations of distinct subunits whose assembly is finely tuned in the ER [104]. PrP interacts physically with the NR1 and NR2D subunits of NMDARs and the GluA1 and GluA2 subunits of the AMPARs, and these interactions are important for normal neuronal physiology and survival [105–108]. It will be interesting to see whether the cellular trafficking and synaptic localization of NMDARs and AMPARs are impaired in neurons expressing mutant PrP and explore any functional consequence.

## 9. Possible Alterations of Secretory Transport in Nongenetic Forms of Prion Disease

The evidence that misfolding of mutant PrP in the ER affects synaptic transmission by impairing membrane delivery of VGCCs raises the question whether a similar mechanism is operative in nongenetic forms of prion disease. In sporadic prion diseases, PrP^C^ is believed to misfold spontaneously at a low frequency. This could preferentially occur during biosynthesis in the ER lumen, where the oxidative folding of the nascent PrP^C^ polypeptide may be affected by perturbations of ER homeostasis. Consistent with this, treatment of neuroblastoma N2a cells with several ER stressors caused the formation of a misfolded PrP^C^ isoform that was more prone to PrP^Sc^ conversion [109].

In prion diseases acquired by infection, exogenous PrP^Sc^ induces conversion of PrP^C^ on the cell surface or within an endocytic compartment, rather than in the ER [15–17]. However, stimulation of PrP^C^ retrograde transport toward the ER increases PrP^Sc^ levels in prion-infected N2a cells, suggesting that ER-localized PrP^C^ may also misfold [110]. In addition, PrP^Sc^ replication perturbs ER calcium homeostasis [111], and this could favor misfolding of newly synthesized PrP^C^ [109]. Thus, several mechanisms may trigger misfolding and ER retention of PrP^C^ in nongenetic prion diseases, potentially interfering with secretory transport of VGCCs and perhaps other PrP-interacting proteins. Intriguingly, VGCC activity is impaired in scrapie-infected GT1 cells [112], but whether this is due to defective transport of the channel to the plasma membrane remains to be established.

Although we have emphasized the role of PrP^C^ misfolding in the neuronal ER, protein trafficking may also be impaired by the accumulation of PrP in other compartments of the secretory pathway. We did in fact find that PrP carrying the FFI mutation accumulates in the Golgi of N2a cells and that its expression is associated with an alteration of the GDI/Rab11 pathway governing post-Golgi vesicular trafficking [113]. A recent report indicates that post-Golgi trafficking is also impaired in prion-infected N2a cells [114].

Finally, misfolded PrP accumulation may alter the secretory transport of PrP-interacting proteins also in nonneuronal cells. For example, PrP^C^ interacts with the *α*2 and *β*2 subunits of Na^+^/K^+^-ATPase in glial cells, and this interaction is involved in regulating glutamate-dependent release of lactate from astrocytes [105]. Lactate released from astrocytes is taken up by neurons and is an important energy source, at least during high neuronal activity. Thus, any impairment of *α*2/*β*2-ATPase transport in mutant or prion-infected astrocytes could contribute to neuronal dysfunction.

## 10. From Synaptic Dysfunction to Neuronal Death: Role of Intracellular PrP Retention in the Phenotypic Heterogeneity of Prion Diseases

Does intracellular PrP accumulation ultimately lead to neuronal cell death? Persistent UPR in the hippocampus of RML-infected mice might kill neurons through activation of the ATF4/CHOP apoptotic pathway [80]. However, degenerating hippocampal neurons in these mice do not show morphological features of apoptosis, suggesting that this may not be the actual effector mechanism of cell death [80]. In addition, PERK and eIF2*α* are not activated in brains of individuals with sporadic, infectiously acquired, or genetic prion disease, not even in those brain areas with the most pronounced neuropathological changes [115].

**Figure 4:**
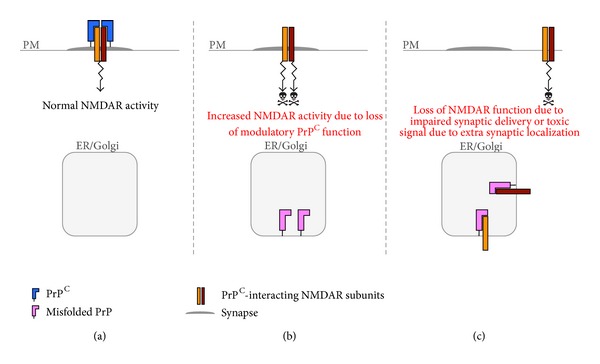
A role for intracellular PrP retention in NMDAR dysfunction. (a) PrP^C^ on the plasma membrane (PM) attenuates NMDAR activity by associating with the NR2D subunit. (b) Owing to PrP^C^ misfolding in transport organelles (ER/Golgi), PrP^C^ is retained intracellularly. This results in increased NMDAR activation, potentially triggering neurotoxicity. (c) Intracellular retention of misfolded PrP^C^ with NR2D and NR1 subunits results in impaired delivery of NMDARs to the cell surface or their abnormal targeting to extrasynaptic sites, leading to loss of NMDAR function and/or activation of neurotoxic stimuli.

**Figure 5:**
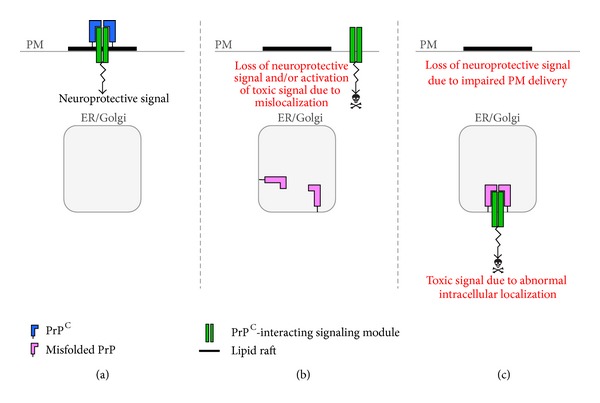
Theoretical model for how intracellular retention could perturb PrP^C^-dependent signaling. (a) PrP^C^ acts as scaffold molecules that keep a prosurvival signaling complex in lipid rafts of the plasma membrane (PM). The lipid raft localization would be essential to activate neuroprotective signaling. (b) Owing to retention in transport organelles (ER/Golgi), PrP^C^ function is lost and the signaling complex localizes in nonraft regions of the PM, losing its neuroprotective activity and potentially eliciting a neurotoxic signal. (c) Misfolded PrP sequesters the signaling module in intracellular compartments, leading to loss of neuroprotective function on the cell membrane. Intracellular retention might also cause the complex to function abnormally and generate a toxic signal.

We would like to offer an alternative explanation for how intracellular accumulation of misfolded PrP might kill neurons. We propose that neuronal death in prion diseases may result from a functional perturbation of proteins that physiologically interact with PrP^C^, either because of sequestration in transport organelles or because their normal activity on the cell surface is corrupted in the absence of PrP^C^ (Figures 4 and 5).

In addition to *α*_2_ *δ* subunits, whose functional impairment could lead to apoptotic cell death [116, 117], other PrP-interacting proteins whose abnormal function could mediate neurotoxic effects are the glutamate receptors. PrP^C^ attenuates activation of NMDARs through its interaction with the NR2D subunit, thereby protecting neurons from glutamate-induced excitotoxicity [106] (Figure 4(a)). This neuroprotective function could be lost with intracellular PrP retention, making neurons more susceptible to excitotoxic stimuli (Figure 4(b)). In addition, misfolded PrP could sequester NR2D or NR1 (the other PrP^C^-interacting NMDAR subunit [108]) in transport organelles, reducing NMDAR plasma membrane delivery or interfering with their correct targeting to the synaptic membrane (Figure 4(c)). This could also result in neuronal damage, since synaptic NMDAR activation promotes survival, while activation of extrasynaptic NMDAR signals causes stress and death [118]. Intracellular retention of GluA1 and GluA2 with misfolded PrP [105, 107] might also be involved. For example, sequestration of GluA2 may result in AMPARs lacking this subunit, which are more permeable to calcium, potentially exacerbating excitotoxic phenomena [119]. Consistent with a role of excitotoxicity in PrP-mediated neurodegeneration, a neurotoxic mutant PrP was recently seen to sensitize neurons to glutamate-induced cell death [120].

Retention of misfolded PrP in the secretory pathway might also indirectly affect PrP^C^-mediated signaling functions. There is increasing evidence, in fact, that PrP^C^ serves as a cell surface scaffold for a variety of signaling modules that control neuronal differentiation and survival [121] (Figure 5(a)). These prosurvival signals may be lost or corrupted in case of misfolding and intracellular retention of PrP^C^, eventually triggering neuronal death (Figures 5(b) and 5(c)). Thus, prion disease pathogenesis may result from toxic activities engaged by intracellular PrP^C^ misfolding in conjunction with loss of PrP^C^ function on the cell surface.

Could this model of toxicity explain the heterogeneous clinical presentation of prion diseases? Ion channels, glutamate receptors, and signaling complexes are generally made of different subunit isoforms, which are expressed in functionally distinct neurons of the brain. PrP^C^ may preferentially interact with specific isoforms, inducing functional abnormalities only in certain types of neurons. For example, PrP^C^ co-immunoprecipitates with the NR2D but not the NR2B subunit of NMDARs [106]. Therefore, PrP^C^ misfolding may specifically affect neurons expressing NR2D.

Then too, different misfolded variants of PrP may differ in their interacting properties. PG14 and D178N PrPs are structurally different [122] and have different ability to interact with the GluA2 subunit of AMPARs [107]. PG14 co-immunoprecipitates with GluA2 as does wild-type PrP, whereas D178N PrP does not [107]. This suggests that intracellular retention of PG14, but not D178N PrP, may impair GluA2 trafficking. Thus, different misfolded forms of PrP may have different effects on neuronal function—hence on the clinical presentation of disease—depending on whether they lose or maintain the ability to interact with their molecular partners.

## 11. Summary and Conclusions

PrP^C^ misfolding has long been known to play a key role in the pathogenesis of prion diseases, but only recently have we started elucidating the neurotoxic mechanisms. Experimental studies have indicated a dissociation between prion infectivity and neurotoxicity, and the assumption that PrP^Sc^ is both infectious and pathogenic is being progressively replaced by the view that noninfectious PrP species are the actual neurotoxic culprits [25, 123, 124]. There is also a great deal of experimental data against the idea that extracellular aggregates of misfolded PrP are intrinsically neurotoxic, indicating instead that neuronal degeneration is triggered by conformational conversion of endogenous PrP^C^ [79, 125–127]. Finally, synaptic dysfunction is emerging as the primary determinant of neurological illness, so therapeutic interventions should aim at preventing synaptic damage, in addition to blocking neuronal death.

The observation that several mutant PrPs acquire abnormal conformations soon after synthesis in the ER and are delayed in their biosynthetic maturation and secretory transport suggested that intracellular accumulation could be crucial in neuronal dysfunction [25]. This is now supported by our demonstration that mutant PrP impairs the synaptic delivery of VGCCs through a physical interaction with *α*_2_ *δ*-1 in transport organelles, leading to alterations in neurotransmission [82]. In this review, we argue that other channels or signaling complexes could gain neurotoxic functions because of misfolding and retention of mutant PrP in the secretory pathway and that similar mechanisms may also be operative in nongenetic prion diseases. The neurotoxic modality that we propose might also explain the clinical heterogeneity of prion diseases, since different pathological conformations of PrP may selectively impair the trafficking and activity of different proteins, preferentially expressed in specific types of neurons.

In conclusion, emerging evidence points to a key pathogenic role of PrP^C^ misfolding in the secretory pathway. Impairment of secretory protein trafficking may be a major cause of neuronal dysfunction and degeneration in prion diseases and perhaps in other neurodegenerative disorders caused by intracellular accumulation of misfolded proteins.
